# Drugging Hedgehog: signaling the pathway to translation

**DOI:** 10.1186/1741-7007-11-37

**Published:** 2013-04-15

**Authors:** Tom J Carney, Philip W Ingham

**Affiliations:** 1 Institute of Molecular and Cell Biology, 61 Biopolis Drive, Proteos, Singapore 138673

## Abstract

First discovered in *Drosophila*, the Hedgehog signaling pathway controls a wide range of developmental processes and is implicated in a variety of cancers. The success of a screen for chemical modulators of this pathway, published in 2002, opened a new chapter in the quest to translate the results of basic developmental biology research into therapeutic applications. Small molecule pathway agonists are now used to program stem cells, whilst antagonists are proving effective as anti-cancer therapies.

## 

In November 2002, in what was only the second research article to be published by the *Journal of Biology*, Jeff Porter and colleagues described their use of a cell-based assay to identify and characterize small molecules that modulate the activity of the Hedgehog signaling pathway [1]. In the preceding decade there had been an explosion of interest in this pathway, stimulated by the discovery of the vertebrate homologues of the *hedgehog *(*hh*) gene that had originally been studied in *Drosophila*. A burgeoning body of evidence supported the conviction that manipulation of Hh signaling could have applications in both regenerative medicine and cancer therapy. The Frank-Kamenetsky *et al*. paper [1], along with a contemporaneous publication from the Beachy group [2], provided a breakthrough in highlighting Smoothened (Smo), the G-protein coupled receptor (GPCR)-like protein that sits at the heart of the pathway (Figure 1), as a highly 'druggable' target. Since this seminal publication, the screening approach as well as the molecules it identified have been extensively exploited: chemical modulators of the Hedgehog pathway, and in particular of Smo, have provided versatile tools in elucidating the mechanism of action and roles of Hh signaling as well as in the development of novel cell replacement and anti-cancer therapies. Here we reflect on the significance of the Frank-Kamenetsky paper at the time of its publication and the impact that chemical modulators of Hh pathway activity have had over the succeeding decade.

**Figure 1:**
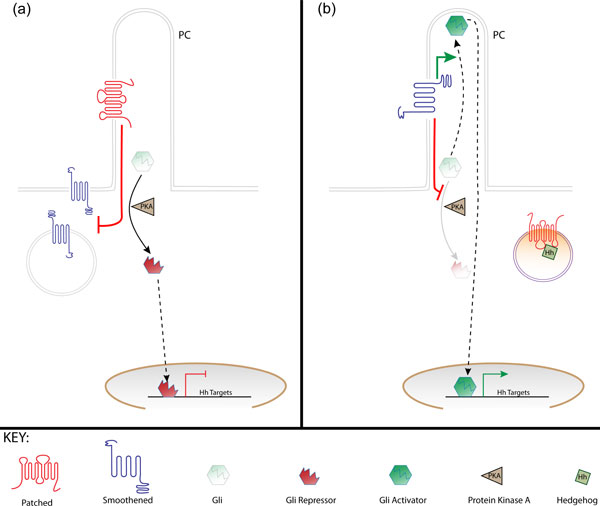
**Hedgehog pathway overview**. In vertebrates, the response of cells to Hh ligands is coordinated at the primary cilium (PC), a finger-like projection on the cell surface. **(a) **In the absence of ligand, the Hh receptor Patched localizes to the PC, keeping membrane targeted Smoothened out and in an inactive state. In the absence of Smoothened activity, Protein Kinase A (PKA) localized at the base of the PC promotes the cleavage of the Gli transcription factor into a repressor form that enters the nucleus and represses target gene expression. **(b) **On binding to Hh, Patched is internalized and targeted to the lysosome. Released from its Patched-mediated inhibition, Smoothened moves into the primary cilium where its activity attenuates the PKA-dependent cleavage of the Gli transcription factors, allowing their full-length forms to enter the cilium. Here they are activated by Smoothened before moving to the nucleus to activate target gene transcription.

The cloning of Sonic Hedgehog (Shh), one of three vertebrate Hh orthologues, in 1993 [3-5] led quickly to the discovery of its roles both in patterning the digits of the developing limb and in specifying cell identity in the neural tube. The identification of Shh as the mediator of the so-called 'zone of polarizing activity', the region of the developing limb bud that specifies digit type and number, had the most immediate impact, but it was its function in the developing central nervous system that attracted the attention of those with an eye for clinical application. For here was a protein that potentially could be used to direct the differentiation of neural progenitors into specific cell types, a Holy Grail of the newly defined field of Regenerative Medicine [6]. In a succession of papers, principally from the Jessell laboratory [7], Shh was shown to act in a concentration-dependent manner to induce the specification of distinct types of neurons, opening up the prospect of generating pure populations of differentiated cells for engraftment into patients with neurodegenerative disease.

Such a use of Shh had in fact been anticipated with its original discovery and a patent application relating to its exploitation 'to generate and/or maintain an array of different vertebrate tissue both *in vitro *and *in vivo*' had been filed at the end of 1993 [8]. This patent provided part of the intellectual property upon which the small biotech startup company Ontogeny was founded, with the Hh pathway a central focus of its activities. In a matter of months Hh signaling had moved from being the esoteric preserve of a handful of *Drosophila *geneticists to a potential target for pharmaceutical manipulation. And since pharmaceutical companies prefer the lower production costs and longer shelf life of small molecules compared to biologics, the search began for chemical modulators of its activity.

The early studies of Hh signaling in *Drosophila *had identified the multipass-transmembrane protein Patched as being the receptor for Hh [9] and the GPCR-like protein Smoothened as an obligate transducer of its activity into the cell [10,11]. GPCRs are much favored by the pharmaceutical industry, being recognized as highly 'druggable' targets by virtue of their cell surface location and many examples exist of small molecule GPCR agonists and antagonists [12]. So the prospects for identifying chemical modulators of the Hh pathway seemed good. In fact, a precedent for such a modulator had been established in 1998, with the demonstration that the naturally occurring alkaloid, cyclopamine, acts as a specific inhibitor of the Hh pathway [13].

Encouraged by this finding, Porter and colleagues at Ontogeny (which in 2000 merged with two other companies to become Curis Inc.) set out to discover novel chemical modulators of Hh. Up to this point, *in vitro *assays of Hh pathway activation had been based either on the differentiation of explanted embryonic tissues or on alkaline phosphatase activity in 10T1/2 cells, an assay that took over 5 days to complete [14]. The key to the success of the Curis screen was the development and optimization of a rapid, high-throughput cell-based assay using an easily quantifiable Hh-sensitive luciferase reporter. Studies in *Drosophila *of the cryptically named Cubitus Interruptus protein, a member of the Gli family of transcription factors, had revealed its role in mediating the transcriptional response to Hh through binding to enhancers upstream of *ptc*, itself a Hh target gene [15], a finding that provided the basis for the development of such a Hh-reporter. 10T1/2 cells stably transfected with a luciferase reporter gene driven by multimerized Gli binding sites responded strongly to addition of exogenous Shh, permitting screens both for inhibitors (in the presence of Shh) and activators (in the absence of Shh). Beginning in late 1999, the Curis group used this approach to screen a library of 140,000 compounds and, as anticipated, identified both agonists and antagonists of the pathway. Amongst the latter was CUR-61414, a potent Smo inhibitor the characteristics of which would be explored in depth in subsequent studies. The principal focus of the Frank-Kamenetsky *et al*. paper, however, was the identification of Hh-Ag1.1, the first example of a Hh-pathway agonist.

By synthesizing hundreds of Hh-Ag1.1 derivatives, several related compounds (Hh-Ag 1.2, 1.3, 1.4 and 1.5) were identified with enhanced potency, reduced toxicity and/or improved stability. Not only was the strong transcriptional activation evoked by these agonists reproducible *in vivo*, they also mimicked the cellular responses elicited by Hh proteins, acting as potent mitogens on primary rat cerebellar neurons and inducing specific neuronal progenitor subtypes in neural plate cultures. This recapitulation of Hh neural patterning activity together with the ability to rescue Shh mutant embryos by oral gavage of pregnant mothers established the *in vivo *utility of the Hh-Ag molecules. A set of epistasis tests suggested that both agonists and antagonists (cyclopamine and CUR-61414) act at the same level downstream of the Hh receptor Patched, but upstream of the intracellular modulator of Gli activity, Protein Kinase A (PKA). Moreover, the inability of agonists to rescue the mouse *Smo *mutant phenotype pointed to the Smo protein itself being their target. Formal proof of this came through use of radiolabeled agonist in Smo protein binding and competition assays, which at the same time proved that the antagonists also act by binding Smo directly (see Figure 2). The analysis of Hh-Ag1.3 by the Beachy group, who coined the name SAG (Smoothened AGonist) for this particular molecule [2], confirmed these findings.

**Figure 2:**
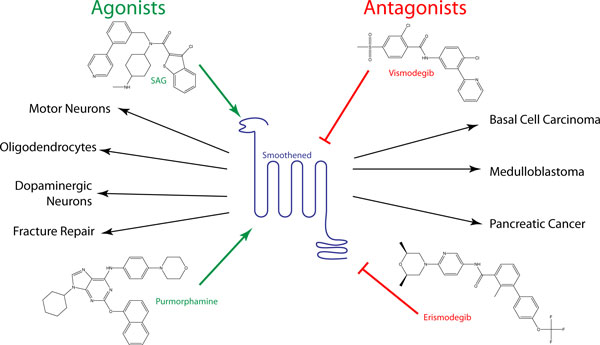
**Small molecule manipulation of the Hh pathway**. Major small molecule agonists (left) and antagonists (right) of Smoothened (center), and their clinical uses. Antagonists such as vismodegib (GDC-0449) and erismodegib (LDE225) are currently being trialed against a number of Hh pathway-activated cancers, whilst the two main agonists, SAG (Hh-Ag1.3) and purmorphamine, are being used for directed differentiation of stem cells to a variety of cell types.

The first reported application of SAG in directing differentiation of embryonic stem (ES) cells actually pre-empted publication of the Frank-Kamenetsky *et al*. paper. In the August 2002 edition of *Cell*, Jessell and colleagues [16] reported that treatment of mouse ES cells with SAG in combination with retinoic acid treatment could induce the formation of functional motor neurons. Since then, there have been many other reports of the directed differentiation of stem cells through the manipulation of the Hh pathway, using both SAG as well as other pathway agonists, notably purmorphamine. Interestingly, this compound, also first described in 2002, was originally identified through its capacity to induce mesenchymal progenitor cells to differentiate into osteoblasts and only subsequently shown to be an Hh pathway agonist that, like SAG, binds directly to Smo [17] (Figure 2).

The use of purmorphamine to generate oligodendrocytes from ES cells provided the first demonstration that Hh agonists can induce glial as well as neuronal differentiation *in vitro *[18]. Excitingly, Goldman and colleagues have now described the successful amelioration of symptoms in a murine model of congenital hypomyelination through engraftment of oligodendrocyte progenitor cells derived in this way from human induced pluripotent stem cells [19]. Both purmorphamine and SAG have also been used to promote the induction of dopaminergic neurons (DA), the loss of which is the principal cause of Parkinson's disease. In a striking example of this application, Studer and colleagues showed that human ES cell-derived DA neurons were sufficiently stable to support long-term engraftment in several different lesioned animal models, resulting in significant recovery of motor function in each case [20]. In another ground-breaking study, Sasai and colleagues used SAG to induce formation of three-dimensional, functional pituitaries from ES cell-derived ectodermal co-cultures [21]. Together, these remarkable studies underline the efficacy of Hh pathway agonists in programming human stem cells and hold out great promise for development of effective cell-based therapies in the not too distant future.

As well as its role in neural patterning and survival, the Hh pathway functions in a variety of other developmental processes. The ability of purmorphamine to direct mesenchymal cells to an osteogenic fate has been shown to occur via induction of the master regulator of bone formation, Runx2, and there has already been an attempt to load purmorphamine onto artificial bone adhesives to promote fracture repair [22]. In mouse skin, topical application of SAG has also been used to stimulate hair follicle entrance into anagen phase, potently enhancing hair regrowth [23]. Additionally, SAG has been shown to ameliorate cortisol-mediated neurotoxicity in cerebral granule neuron precursors by restoring Hh signaling levels, suggesting its potential as a neuroprotective agent for glucocorticoid-induced neonatal cerebellar injury [24]. Similarly, in an *in vitro *model of ALS, purmorphamine was shown to be cytoprotective against oxidative stress [25]. Together, these findings raise the possibility that Hh agonists could be exploited in the clinic as neuroprotective agents.

Clinical application of Hh pathway antagonists (Figure 2), by contrast, is already a reality and represents an equally, if not more, significant impact of the Porter paper. The discovery of the link between loss of Patched function in individuals suffering from Gorlin's syndrome [26,27] and their susceptibility to basal cell carcinomas (BCCs) set in train a rapid exploration of the role of Hh pathway dysfunction in tumorigenesis. By the beginning of the new millennium, Hh signaling had been implicated in a significant number of cancers, including lung, prostate and pancreatic [28] as well as BCC and medullobalstoma, the most prevalent and recalcitrant form of brain tumor in children [29]. The identification of the small molecule Hh inhibitor CUR61414 reported in the *Journal of Biology *paper was followed up in early 2003 with a report of the first evidence for therapeutic efficacy of such a molecule. Two *in vitro *BCC culture systems were established by the Curis team, in both of which CUR61414 was found to elicit complete regression of the lesions [30]. The following year Curran and colleagues reported the successful elimination of medulloblastomas in *Ptc1/+; p53/p53 *mice, following treatment with HhAntag-691, another antagonist from the Curis screen [31]. Within five years, the first reports of therapeutic efficacy in humans of a more potent derivative, GDC-0449, were published: in one case, transient regression of a metastatic medulloblastoma was observed in a patient who failed to respond to other therapies [32], whilst in the second case over 50% of a cohort of patients with metastatic BCC were reported to respond favorably to oral dosing with GDC-0449 [33]. These promising results prompted further phase 2 and 3 clinical trials [34] and in early 2012, GDC-0449, now known as vimodegib or erivedge, received Food and Drug Administration approval as a therapy for metastatic BCC.

While many challenges remain, not least emergence of mutant forms of Smo that are resistant to vismodegib, the prospects for the use of Hh antagonists as therapies for a range of cancers look promising. By any measure, the development of a novel anti-cancer drug as well as reagents promoting the directed differentiation of human stem cells represents an impressive output from a single screen!

## Note

This article is part of the BMC Biology tenth anniversary series. Other articles in this series can be found at http://www.biomedcentral.com/bmcbiol/series/tenthanniversary.
